# Method for Detecting Manipulated Compilation of Sensing Reports in Wireless Sensor Networks

**DOI:** 10.1155/2015/493162

**Published:** 2015-05-03

**Authors:** Hae Young Lee

**Affiliations:** Department of Information Security, Seoul Women's University, 621 Hwarang-ro, Nowon-gu, Seoul 139-774, Republic of Korea

## Abstract

In cluster-based wireless sensor networks (WSNs), a few sensor nodes, including cluster heads (CHs), can be physically compromised by a malicious adversary. By using compromised CHs, the adversary can intentionally attach false message authentication codes into legitimate sensing reports in order to interrupt reporting of the real events. The existing solutions are vulnerable to such a type of security attacks, called *manipulated compilation attacks* (MCAs), since they assume that CHs are uncompromised. Thus, the reports manipulated by compromised CHs will be discarded by forwarding nodes or rejected at base stations, so that real events on the fields cannot be properly reported to the users. In this paper, the author proposes a method for the detection of MCAs in cluster-based WSNs. In the proposed method, every sensing report is collaboratively generated and verified by cluster nodes based on very loose synchronization. Once a cluster node has detected an MCA for a real event, it can reforward a legitimate report immediately. Therefore, the event can be properly reported to the users. The performance of the proposed method is shown with analytical and experimental results at the end of the paper.

## 1. Introduction

A wireless sensor network (WSN) [1] consists of a large number of small sensor nodes reporting real events (e.g., the appearances of vehicles) on the field and a few base stations (BSs) that collect sensing reports of the nodes. In many applications, these nodes are deployed in hostile environments, such as battlefields, and not attended by the users, so that a malicious adversary can physically capture some of them and thus potentially compromise the whole of their information, including keying material [2]. By using such compromised nodes, the adversary can inject* fabricated sensing reports*, which represent nonexistent events in the field, into the network with the goal of deceiving the BSs or depleting the limited energy resources of forwarding nodes [3, 4]. To prevent them from fabricating reports, every sensing report should be endorsed by multiple nodes, through attaching multiple message authentication codes (MACs) generated by them using different cryptographic keys, as in [3–8].

On the other hand, such a collaborative process has given rise to another security vulnerability in which some comprised nodes can intentionally attach false MACs to legitimate reports in order to interrupt reporting of the real events [8]. Although several security solutions [9–12] have been recently proposed to deal with such a type of security attacks, called* false-endorsements insertion attacks* (or* false-endorsement-based denial-of-service (DoS) attacks*) (FEIAs), they have a common vulnerability; a real event cannot be reported if a delegate, usually a cluster head (CH), for the event is compromised since they assume that the node is honest.

**Figure 1:**
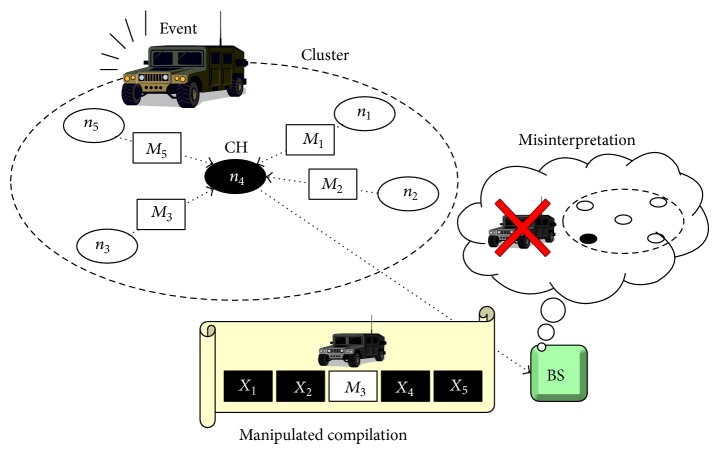
An example of manipulated compilation attacks (MCAs).

Let us suppose a cluster having a compromised CH, *n*_4_, as shown in Figure 1. When *n*_4_ has detected an event, it first announces the detection of the event. Once the announcement has been accepted by the other nodes in the cluster, *n*_1_, *n*_2_, *n*_3_, and *n*_5_, they send legitimate MACs, generated using their keys, for the event to the CH, in order to endorse the announcement. Although *n*_4_ has collected a large enough number of legitimate MACs (i.e., *M*_1_, *M*_2_, *M*_3_, and *M*_5_), it can, in order to suppress reporting of the event, attach false MACs (*X*_1_, *X*_2_, *X*_4_, and *X*_5_) to a report or modify the contents of the report or even discard the report. Such a report would be filtered out by a forwarding node since the node considers the report to be a fabricated one. Moreover, if the report is delivered to a BS, innocent nodes could be misinterpreted as compromised ones. For example, in Figure 1, the BS could consider the report to be a fabricated one injected by a node *n*_3_, which might be compromised, since every MAC in the report is incorrect except *M*_3_.

In this paper, the author proposes a method for detecting such a type of security attacks, called* manipulated compilation attacks* (MCAs), in cluster-based WSNs. In the proposed method, for an event, a report is collaboratively generated by nodes in a cluster and forwarded by the CH, as in the existing solutions. In contrast to them, the method allows the report to be verified by the other nodes in the same cluster; if one of them has detected an MCA, the node can reforward a legitimate report generated by it. Therefore, the event can be properly reported to the users. In order to facilitate such a collaborative verification process, the local clocks of nodes in a cluster are very loosely synchronized while each report is being generated and verified. The performance of the proposed method is analyzed by analytical and experimental methods.

The remainder of the paper is organized as follows: Section 2 introduces the proposed method in detail, under the assumption of an ideal environment.  Section 3 reviews the results of the performance analysis on the proposed method. In Section 4, some considerations for applying the proposed method to real-world WSNs are discussed. Related work on MCAs is surveyed in Section 5. Finally, conclusions and future work are then discussed in Section 6.

## 2. Detection of Manipulated Compilation Attacks (MCAs)

In this section, the proposed method is described in detail, under the assumption of an ideal environment. Some considerations for real-world applications are discussed in Section 4.

### 2.1. Network Model

A highly dense cluster-based WSN is considered since dense WSNs are resilient against false data injection attacks (FDIAs) [3–8], in which malicious adversaries inject fabricated sensing reports into the networks using a few compromised nodes with the goal of energy draining and/or false alarms. It is assumed that sensor nodes are similar to the current generation of sensor nodes (e.g., 802.15.4 Motes produced by [13]), in terms of computational and communication capabilities, and energy resources. When an event is detected by a node in a cluster, it is also detected by the other nodes in the cluster simultaneously. All nodes in a cluster can directly communicate with each other. A message transmitted by one of them can be heard by the other nodes in the cluster due to the broadcast nature of the wireless communications. Each node has some keys shared with the BSs and some other keys shared with all nodes in the same cluster. Some of the former keys can be shared with forwarding nodes in order to facilitate en-route verification (e.g., by [9] or [10]) of sensing reports. The latter keys are used to verify the origins of messages within the cluster.

### 2.2. Threat Model and Design Goal

It is assumed that a malicious adversary can physically compromise a few nodes, including CHs. However, the adversary cannot compromise the BS and half of the nodes in each cluster without being detected. The goal of the adversary is to suppress reporting of real events on the field, so that the adversary uses compromised nodes to launch MCAs against real events. The goal of the proposed method is to detect such misbehavior without any special equipment. Even if an MCA has been launched against a real event, a legitimate report for the event must be restored by honest nodes in order to report the event to the users. The method should be simple enough to be implemented on low-end node devices. It is also assumed that compromised nodes cannot forge their identities; such forgeries can be detected by using [14]. Please note that the identification of compromised nodes (e.g., as in [15]) is a separate issue and beyond of the scope of this paper.

### 2.3. Basic Procedure

**Figure 2:**
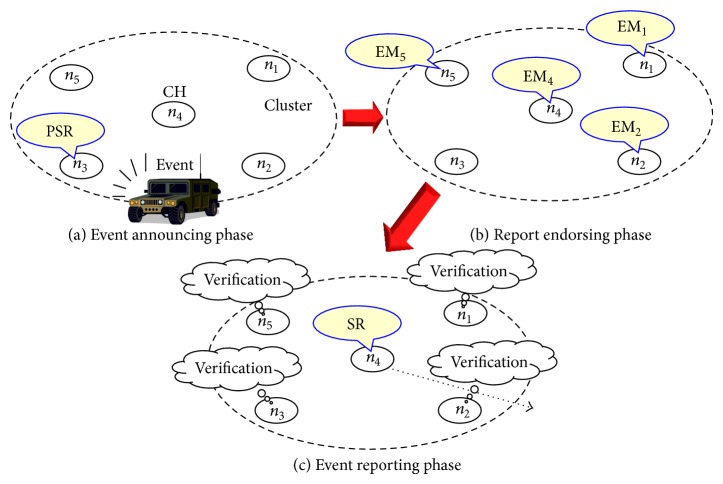
Basic procedure of the proposed method.

Figure 2 shows the basic procedure of the proposed method. In the method, every sensing report is generated through the three phases: (a)* event announcing phase*, (b)* report endorsing phase*, and (c)* event reporting phase*. In the event announcing phase, a node in a cluster announces the detection of an event to the other nodes in the same cluster. If the nodes accept the announcement, their local clocks are very loosely synchronized by the receiving of the announcement. In the report endorsing phase, the nodes send messages to endorse the announcement to the CH. Due to the broadcast nature of wireless communications, all the nodes in the cluster can collect these endorsement messages. Finally, a report is forwarded by the CH and verified by the other nodes in the cluster within the event reporting phase. If one of them finds an MCA, it reforwards a report prepared by it. Thus, the event would be properly reported to the users. Please note that other loose synchronization methods [16, 17] or channel access methods (e.g., FDMA) could be used for very prompt notifications of events (see Section 4.1).

### 2.4. Event Announcing Phase

When a node in a cluster detects a real event, the event is also detected by the other nodes in the same cluster simultaneously. Each of the detecting nodes in the cluster (i.e., all the nodes in the cluster) then prepares a* preliminary sensing report* (PSR) for the event and sets a random timer. The PSR would contain the ID of the node, the contents for the event, and two MACs over the contents, generated using two of the node's keys, one for en-route verification and another for source verification. Upon the expiration of the timer, the node broadcasts its PSR and then resets its local clock to 0. When one of the other detecting nodes has received the PSR, the node checks if the PSR states the same event detected by the node and was generated by one of the nodes in the cluster. If so, the node accepts the PSR. Once a node in the cluster has accepted the PSR, the node cancels its timer and resets its local clock to 0. Therefore, if all the nodes in the cluster accept the PSR, their lock clocks are very loosely synchronized.

Each of the accepting nodes then prepares an endorsement message (EM), usually containing the ID of the node and two MACs over the contents, generated using two of the node's keys (as in PSR). A compromised node may not broadcast a PSR, in order to suppress reporting of the event. However, the detection of the event will be announced by an honest node in the cluster, so that the process will continue. From the synchronization point (upon accepting the PSR), time is divided into 2 rounds, each composed of *N* time slots of a fixed size, where *N* is the number of the nodes in the cluster. The first slot of each round is always assigned to the CH, and the rest of the slots are assigned to the other nodes in the cluster. The determination of the slot size, CH selection, and slot assignment are separate issues and beyond of the scope of the paper. We might use some existing solutions for these issues (e.g., [18, 19] for the assignment), possibly with slight modifications.

**Figure 3:**
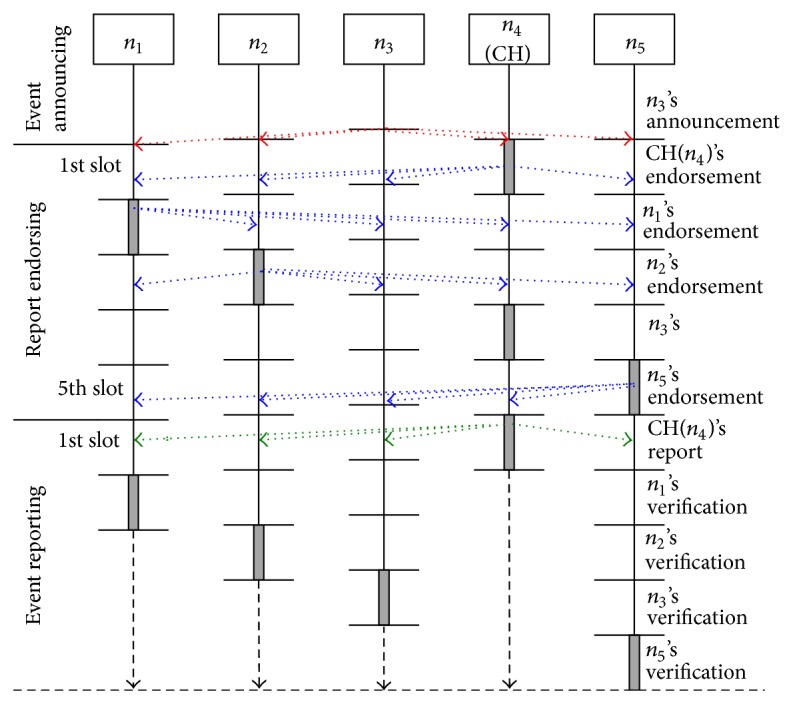
Report generation and verification in the proposed method.

As shown in Figure 3, let us suppose that there is a cluster consisting of 5 nodes, *n*_0_,…, *n*_5_, and *n*_4_ which is the current CH. For an event, *n*_3_ has first broadcasted its PSR (“red” dotted arrows within event announcing), and set its local clock to 0. *n*_1_, *n*_2_, *n*_4_, and *n*_5_ accept the PSR if it states the event detected by them. Then, by setting their local clocks to 0 upon accepting the PSR, the clocks are very loosely synchronized (the uppermost horizontal lines). They also prepare their EMs. Even if a compromised node has not announced the detection of the event, one of the other honest nodes will broadcast a PSR. From the synchronization point, time is divided into 2 rounds, each composed of 5 time slots (i.e., *N* = 5). The first slot of each round has been assigned to the current CH, *n*_4_. The second, third, fourth, and fifth slots of each round have been assigned to *n*_1_, *n*_2_, *n*_3_, and *n*_5_, respectively.

### 2.5. Report Endorsing Phase

In the report endorsing phase, each of the accepting nodes broadcasts its EM, within the slot assigned to it. Meanwhile, every node in the cluster collects these EMs and prepares a sensing report (SR). The SR should include the IDs of the cluster and node, the contents, and *N* MACs collected from the PSR and EMs. Some compromised nodes may not broadcast their EMs. In this case, a node simply puts* blank MACs*, which are filled in 0 s, on the positions of their MACs in the SR. Compromised nodes may also broadcast EMs containing false MACs. However, unless ⌈*N*/2⌉ or more nodes are compromised, ⌈*N*/2⌉ or more legitimate MACs can be collected and attached into the SR.

In the example shown in Figure 3, the current CH, *n*_4_, first broadcasts its EM within the first slot. *n*_1_ and *n*_2_ then broadcast their EMs within the second and third slots, respectively. Although the fourth slot is assigned to *n*_3_, the other nodes have already collected *n*_3_'s MAC from the PSR. Thus, *n*_3_ need not broadcast an EM. Finally, *n*_5_ broadcasts its EMs within the last slot. These broadcasts of EMs are represented in “blue” dotted arrows within report endorsing in the figure. Meanwhile, each of them collects these EMs and prepares an SR. Even if two of the nodes (e.g., *n*_1_ and *n*_2_) are compromised, 3 legitimated MACs (e.g., from *n*_3_, *n*_4_, and *n*_5_) can be collected. If they have not broadcasted their EMs, blank MACs are put on the positions of their MACs in the SR.

### 2.6. Event Reporting Phase

In this phase, the CH forwards its SR on behalf of the other nodes in the cluster, within the first slot. Due to the broadcast nature of wireless communications, theses nodes can also hear the SR forwarded by the CH. Each of them, within its slot, compares the SR lastly forwarded with its SR (the SR prepared by it). If they match exactly, the former is considered to be legitimate. But if not, the former is considered manipulated, so that the node forwards the latter immediately within its slot. The SR newly forwarded can be also verified by the* remaining nodes* that have not compared them yet in the cluster. A compromised CH may forward a SR manipulated, or even nothing. However, such an MCA can be detected by the remaining honest nodes in the cluster. If the last slot has been assigned to a compromised node, no one can verify a SR of the node. Nevertheless, a legitimate SR would have been already forwarded by an honest node in the cluster before the last slot. Therefore, unless ⌈*N*/2⌉ or more nodes are compromised, a legitimate SR, which has ⌈*N*/2⌉ or more legitimate MACs, can be delivered to a BS.

In the example shown in Figure 3, the current CH, *n*_4_, first forwards its SR toward a BS within the first slot of the second round. Within the second slot, *n*_1_ compares the SR forwarded by *n*_4_ with its SR. If they do not match, or the CH did not forward a SR before the second slot, it reforwards the latter immediately toward the BS. In this case, *n*_1_'s SR is also verified by the remaining nodes, *n*_2_, *n*_3_, and *n*_5_, within their slots. Please note that *n*_2_ will verify *n*_4_'s SR within the slot if *n*_1_ has forwarded nothing. If *n*_2_ has found an MCA, or *n*_4_ and *n*_1_ did not forward a SR before the third slot, it reforwards its SR. The remaining nodes, *n*_3_ and *n*_5_, will also verify the lastly forwarded SR. Therefore, unless three or more nodes are compromised, at least one legitimate SR would be forwarded by other honest nodes.

### 2.7. En-Route Verification of Sensing Reports

While a SR is being forwarded toward a BS, forwarding nodes can check the legitimacy of the SR based on a FEIA countermeasure, such as [9]. Blank MACs are considered to be false ones. In order to enable every SR to be delivered to BSs, a centralized detection solution, such as [20], can be applied to the network. However, in general, such solutions cannot provide a mechanism to filter fabricated reports out en-route, so that they can be vulnerable to injecting fabricated reports in terms of energy saving.

## 3. Performance Analysis

The performance of the proposed method is analyzed in this section.

### 3.1. Communication Overhead

The proposed method can be employed to generate sensing reports in the existing FEIA countermeasures [9–12]. In the method, a PSR usually consists of the ID of a node, the contents for an event, and two MACs over the contents generated using a key shared with BSs and another one shared with the other nodes in the same cluster. An EM is usually comprised of the ID of a node and two MACs over the contents generated using two keys. A SR usually includes the ID of a cluster, the ID of a node, the contents for an event, and *N* MACs generated using different keys from different nodes in the cluster. In case of using a global key pool (e.g., when the multipath-based en-route filtering scheme (MEF) [10] is used), the indices of keys used to generate MACs are included in the messages. Also in the existing solutions, messages in a similar format are used to collaboratively generate a report.

The sizes of these messages in bytes are summarized in Table 1, where *S*_CID_ is the size of a cluster ID, *S*_NID_ is the size of a node ID, *S*_*C*_ is the size of the contents for an event, *S*_KID_ is the size of a key ID, and *S*_MAC_ is the size of a MAC. In terms of message sizes, the difference between the method and them is *S*_MAC_ in a PSR.

Both in the method and existing solutions, a report is collaboratively generated through the same number of message transmissions and receptions within a group (e.g., a cluster). Therefore, their communication overhead is

(1) Ocomm=PSR×Otrans⁡+N−1Orecept+EM×N−1Otrans⁡+N−12Orecept+SR×Otrans⁡+N−1Orecept,

where is |*M*| is the size of message *M*, *O*_trans⁡_ is the communication cost for a transmission, and *O*_recept_ is the communication cost for a reception.

### 3.2. Computation Overhead

The computation overhead of the proposed method has been analyzed based on the number of the MAC computations (generation and verification) required to generate a report, as in [3]. In the event announcing phase of the method, at most 2 · *N* MACs are generated by all nodes in a cluster. In case of deterministic announcement (e.g., a preassigned node always announces the detection of an event), 2 MACs are generated by a node. Once a node has broadcasted a PSR, a MAC of the PSR is verified by the other nodes (i.e., *N* − 1 nodes) in the cluster to check the origin of the PSR. In the report endorsement phase, 2(*N* − 1) MACs are generated by them. Each of *N* − 1 MACs attached in EMs is then verified by *N* − 1 nodes to check the origins of the EMs. No MAC is generated or verified in the event reporting phase.

The existing solutions also require a report to be generated through multiple MAC computations: (1) when an event has been detected, at most *N* MACs are generated by all participating nodes before the announcement of the detection. Once the detection has been announced by a node (announcing node), the MAC attached in the announcement is verified by the other participating nodes. In case of deterministic announcements, a MAC is generated by an announcing node and then verified by the other participating nodes. (2) In order to endorse the announcement, a single MAC is generated by the announcing node and 2(*N* − 1) MACs are generated by the other participating nodes. The announcing node collects them and verifies *N* − 1 MACs to check origins of the endorsements. (3) No MAC is computed when the announcing nodes has forwarded a report.

The number of MAC computations is summarized in Table 2, where EAP, REP, and EFP are the event announcing phase, report endorsement phase, and event forwarding phase. In the method, every node in a cluster prepares a SR with the verification of EMs, so that the computation overhead of the method is larger than that of the existing ones, especially in REP. However, the method can provide MCA resilience for WSNs, whereas the others are vulnerable to MCAs.

### 3.3. Energy Efficiency

The energy efficiency of the proposed method has been analyzed based on these communication and computation overheads. It is assumed that *S*_CID_ = 2, *S*_NID_ = 2, *S*_*C*_ = 8, *S*_KID_ = 2, and *S*_MAC_ = 2. It is also assumed that *O*_trans⁡_ = 16.25 *μ*J, *O*_recept_ = 12.5 *μ*J, and 15 *μ*J is consumed for each MAC computation, as in [3]. The amount of energy resources consumed by the generation of a report has been measured. Since SR in the proposed method is identical to that in the existing countermeasures, the energy consumption due to the forwarding of a report is not considered.

**Figure 4:**
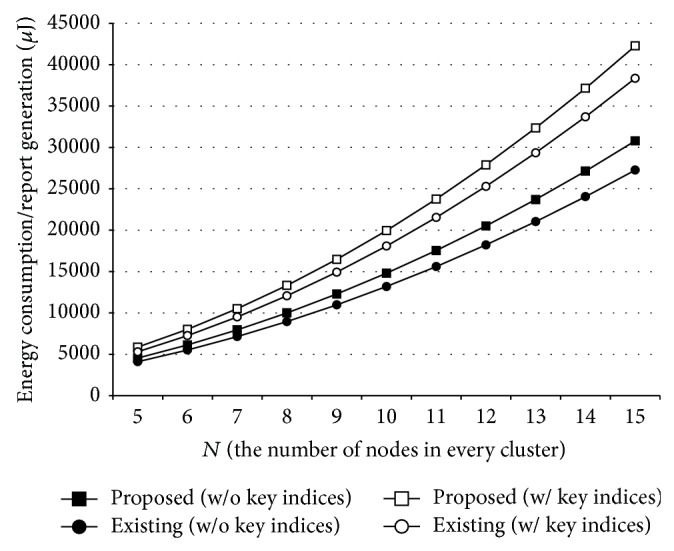
Energy efficiency of the proposed method in case of random announcement.

**Figure 5:**
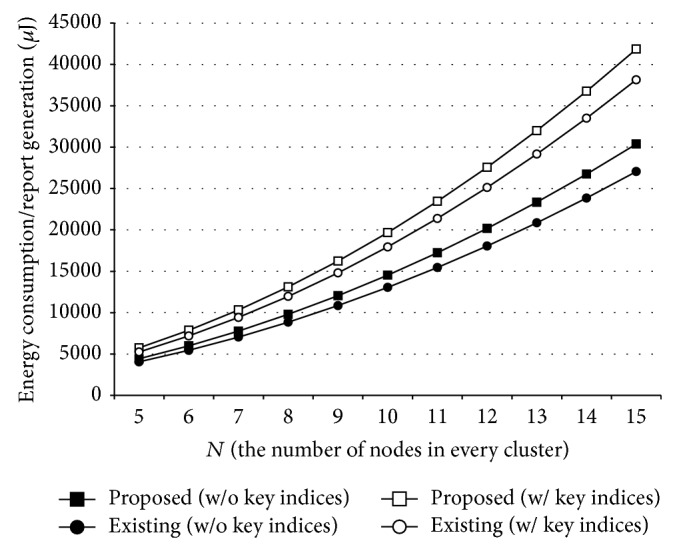
Energy efficiency of the proposed method in case of deterministic announcement.

Figures 4 and 5 show the energy efficiency of the proposed method when *N* is between 5 and 15. A report is generated through a random announcement in Figure 4, whereas an announcing node is deterministically chosen in Figure 5. As shown in the figures, the proposed method (rectangles) consumed more energy resources than the existing solutions (circles) due to the mutual verification among cluster nodes. However, it could provide WSNs with the resilience to MCAs, so that real events on the field could be properly reported to the users. In case of using a global key pool especially, extra energy consumption could be reduced to 9~10%.

### 3.4. Resilience to MCAs

If *C*  (≤*N*) nodes in a cluster have been compromised by an adversary, a SR generated by an honest node for a real event could include at most *C* false MACs in the proposed method. At a BS, a report can be considered to be legitimate if at least half of the MACs attached in the report are correct. Therefore, unless ⌈*N*/2⌉ or more nodes in a cluster are compromised, SRs generated in the cluster will be properly interpreted at BSs.

The resilience of the method against MCAs has been also evaluated through simulation. A field size of 200 × 200 m^2^ is used and a single BS is located at the end of the field. The network consists of 400 clusters and each covers 10 × 10 m^2^. Note that the proposed method deals with in-cluster generation and verification of reports. Thus, it is not virtually affected by the size of the network. In each cluster, *N*/2 − 1 nodes, including the CH, are compromised by an adversary (for the performance evaluation). 2,000 real events occurred on random locations. The method is employed to generate reports in two existing FEIA countermeasures: the probabilistic voting-based filtering scheme (PVFS) [9] and MEF [10].

**Figure 6:**
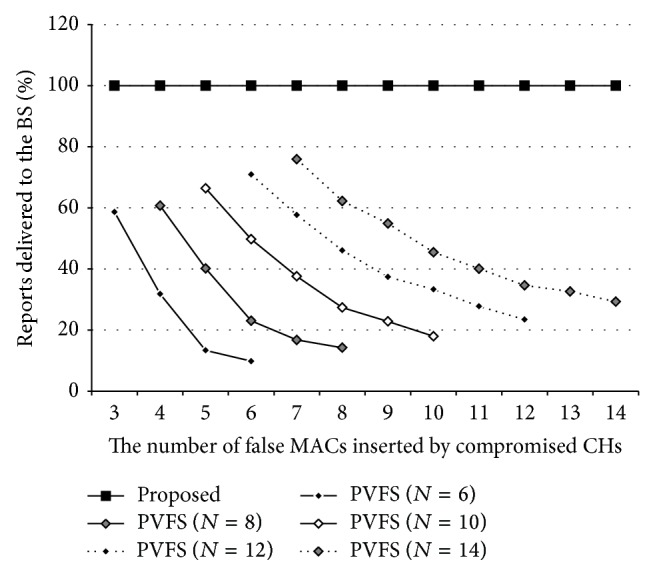
MCA resilience of the proposed method on PVFS.

Figure 6 shows the percentage of the reports delivered to the BS (PRD) on a PVFS-based WSN when *N* is 6,8,…, 14 and the number of false MACs per report, inserted by a CH (i.e., due to an MCA), is between 3 and 14. A forwarding node discards a report if the number of false MACs in the report reaches *N*/2 (i.e., half of them are incorrect). As shown in the figure, the method (filled rectangles) employed for PVFS could guarantee that an event would be always delivered to a BS regardless of MCAs until *N*/2 − 1 compromised nodes since every report would have at most *N*/2 − 1 false MACs. In contrast, PVFS (diamonds) without the method was totally vulnerable to MCAs; even a single compromised CH could make legitimate reports filtered out through the manipulation of their MACs. Moreover, even if such reports could be delivered to the BS, they would be rejected by the BS. Therefore, reporting of the events occurring around the cluster could be completely suppressed by the CH with ease. The method would be enough worth considering since it can provide MCA resilience for the network with 10% overhead.

**Figure 7:**
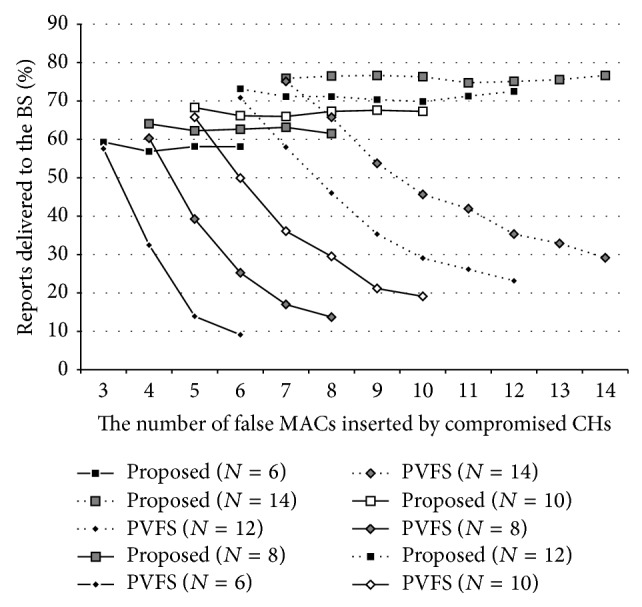
MCA resilience of the proposed method on PVFS when half of the nodes in a cluster are compromised.

Figure 7 shows PRD on a PVFS-based WSN when half of the nodes in a cluster are compromised. As shown in the figure, the method (filled rectangles) could provide the network with MCA resilience to a certain degree. If an event could be detected by two or more clusters (e.g., due to its movement), the event would be properly reported to the users with the method. Please note that the acceptance of them at the BS is another issue. In contrast, the PRD of PVFS severely decreased with the number of false MACs, which might result in a malfunction of the network. Note that reports were discarded by the verification mechanism of PVFS, not by the proposed method. In PVFS (and also in MEF), PRD would decrease as reports travel more hops. Thus, for ultra-large-scale WSNs, we should slightly alter some PVFS parameters to achieve a sufficient level of PRD. However, such alterations also decrease the resilience of the networks against to FDIAs (see Section 5).

**Figure 8:**
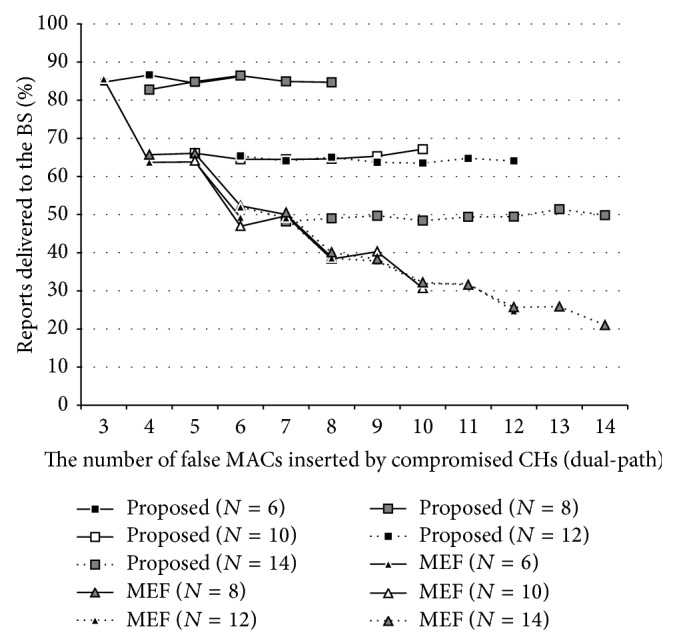
MCA resilience of the proposed method on MEF w/dual-path routing.

Figure 8 shows PRD when MEF using dual-path routing is applied to the network, *N* is 6,8,…, 14, and the number of the false MACs per report is between 3 and 14. There are 2,000 keys in the global key pool maintained by the BS and each node loads 50 keys randomly chosen from the pool. In MEF, a forwarding node drops a report immediately if the report carries any false MAC. Thus, even the method (rectangles), as shown in the figure, could not guarantee that every legitimate report would be delivered to the BS. However, the PDR of the method, while being affected by the number of compromised nodes, was not affected by the number of false MACs inserted by compromised CHs, so that the method is resilient to MCAs. In contrast, the PDR of MEF (triangles) was seriously affected by the number of the false MACs. Also, a report manipulated by a compromised CH, while stating a real event, would be eventually rejected by the BS even if it could be delivered to the BS. Thus, method would be well worth considering despite its overhead.

**Figure 9:**
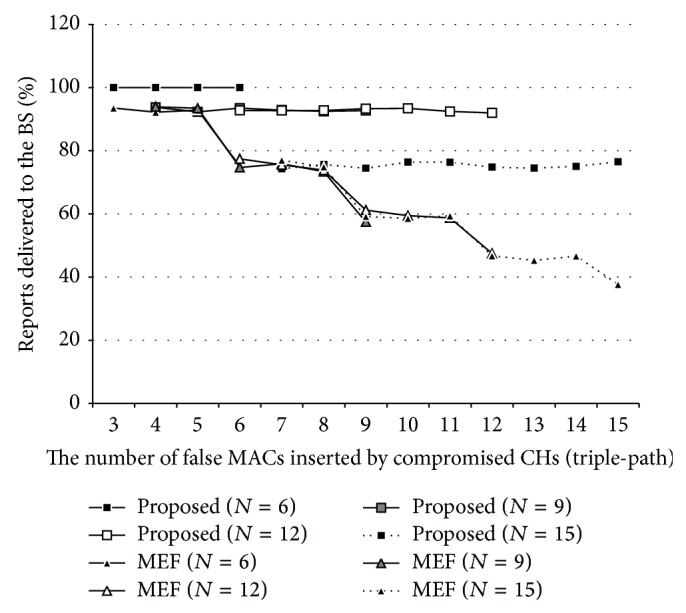
MCA resilience of the proposed method on MEF w/triple-path routing.

Figure 9 shows PRD when MEF using triple-path routing is applied to the network, *N* is 6,9,…, 15, and the number of the false MACs per report is between 3 and 15. As shown in the figure, the PRDs of the method and MEF increased in triple-path routing. The PRD of the method especially (rectangles) was largely enhanced with the routing although more energy resources would be consumed for event reporting. In contrast, the vulnerability of MEF (triangles) was not alleviated regardless of using more energy resources.

## 4. Consideration for Real-World Applications

This section discusses some considerations for applying the proposed method to real-world WSNs.

### 4.1. Collaboration Problems

The proposed method employs a loose time synchronization mechanism for collaborative reporting on events, in which local clocks in a cluster are very loosely synchronized by receiving a PSR. A benefit of such a simple mechanism would be that communication and computing overheads could be reduced, so that it could save energy resources and be implemented on low-end node devices. On the other hand, due to its imprecision in time synchronization, the size of a slot, *T*_*S*_, must be large enough to minimize synchronization errors. That is, its applications must allow a large latency in reporting events. The author argues that such a large latency would be acceptable for real-world responses in large-scale WSNs since it might be much smaller than delays in real-world responses; even *T*_*S*_ = 1 s might be acceptable for ultra-large-scale WSNs.

For some other applications that require very prompt notifications of events, the following approaches could be considered.

- Use of other loose synchronization methods: other loose synchronization solutions [16, 17] that can provide much more precision (e.g., 7.24 *μ*s [16]) could be employed for the collaboration. For example, one of the detecting nodes is elected as a CH in the initial round comprised of *N* slots. In the next round, endorsements for the event are collected. A report for the event is forwarded and verified in the final round. A problem of the use of such precise solutions is that they could involve more communication and computation overheads.
- Use of other channel access methods: we could use alternative channel access methods, such as FDMA, for the collaboration. For example, nodes in a cluster use different channels, so that endorsements could be collected simultaneously through these channels. If one of them finds an MCA on a SR, it reforwards its report through the channel assigned to it. However, due to additional circuitry requirements to dynamically communicate with different radio channels, the cost of sensor nodes is increased, which is contrary to the objective of sensor network systems [21].

### 4.2. Handling Multiple Events

While a report for an event is being generated by nodes in a cluster, another event can be also detected by them, which could interrupt the generation of the report. This problem might be a security vulnerability since malicious adversaries could misuse it as “physical” denial-of-service attacks. The following approaches could alleviate the problem.

- Shortening of *T*_*S*_: a most simple approach might be to shorten *T*_*S*_, enough to handle multiple events. However, it might increase synchronization errors.
- Buffering of events: if another event occurs during the generation of a report, each node stores the event in a buffer. For the generation of a report on the new event, local clocks need not be reset again; the generation is begun with the third round, which leads to the election of a CH. The report, which includes endorsements collected in the fourth round, would be forwarded and verified in the fifth round. A problem of event buffering is that some events might be missed if events occur very frequently in WSNs.
- Prompt notifications of events: we could handle multiple events by making them being reported promptly, which is discussed in Section 4.1. However, it requires high-performance hardware or additional circuitry.

### 4.3. Missing Events

The proposed method assumes that an event can be detected by all nodes in a cluster simultaneously. However, in the real world, each sensor could miss some events, which would lead to the production of SRs with many blank MACs; such SRs might be considered to be fabricated ones. Thus, PRD would decrease with missing (false) MACs, as shown in Figures 6, 7, 8, and 9. This problem could be alleviated with the following approaches.

- Assignment of multiple nodes to each time slot: for very-dense WSNs, a simple (but effective) approach might be to assign multiple nodes to each time slot. For example, we could deploy 2 · *N* nodes to each cluster and assign two nodes to each time slot. When an event has occurred in a cluster, one of the detecting nodes is elected as a CH through the same procedure. For each slot of the first round, one of the two nodes assigned to the slot first broadcasts its EM by the expiration of its (another) random timer. Upon receiving the EM, the other node checks the EM. If the EM is “correct,” the node cancels its timer and quits from participating in the remaining procedure. Then, an SR would be compiled with the EM and verified by the former node. If not, the latter node broadcasts its EM within the slot. Then, the CH would choose a more “trustable” one to endorse its SR. There is still a probability that the whole nodes assigned to a slot could miss events. However, it might be low enough to make every SR carry few or no blank MACs. Theoretically, the probability is (*P*_ME_)^*N*_NPS_^, where *P*_ME_ is a probability of missing events and *N*_NPS_ is the average number of nodes assigned to a time slot.
- Attachment of partial MACs: the problem could be alleviated by allowing a report carry partial MACs. For example, a report is allowed to carry just *N*/2 MACs, excluding blank MACs. This approach requires a slight modification on PVFS or MEF and very-dense WSNs.
- CH-level cooperation with neighboring clusters: another potential approach might be to make CHs cooperate with each other. In many applications, an event can be detected by multiple clusters simultaneously or a gap of time. Thus, if a CH could not collect enough number of MACs, it might cooperate with its neighboring CHs. A single SR might be produced and forwarded by them. The cooperation of clusters is a separate issue and should be investigated in future.

### 4.4. Packet Loss

The proposed method does not consider packet loss (including due to CRC errors) although wireless links are usually unreliable. Loss of some packets might interrupt the generation of reports. The following discusses potential problems of packet loss for each type of message.

- Loss of some PSRs: loss of PSRs might not be a serious problem since any node who has first broadcasted a “correct” PSR would become a CH; although a few PSRs have been lost, each of the remaining nodes has still a chance to broadcast its PSRs. In very-dense WSNs, most PSRs for an event could be lost due to collisions. Such collisions could be minimized by extending the interval of random timers (i.e., the size of a round) or by using other collaboration methods described in Section 4.1.
- Loss of some EMs: we could consider loss of some EMs to be missing events, so that it would cause the production of SRs with many blank MACs. Thus, it could be alleviated with the approaches described in Section 4.3.
- Loss of some SRs: SR loss might not be a significant problem since the “remaining” nodes would attempt to forward their SRs.

## 5. Related Work

Ye et al. [3] first addressed FDIAs in which fabricated sensing reports are injected through a few compromised nodes in order to make false alarms or energy consumption and then proposed the statistical en-route filtering scheme (SEF) as a countermeasure. In SEF, every sensing report must carry a certain number of MACs, generated by different detecting nodes, using keys from different partitions of the global key pool. A report is, while being forwarded toward a BS, verified by some forwarding nodes and discarded immediately if the verification fails. BSs maintain the key pool, so that a report is finally verified by a BS. Thus, unless an adversary has compromised a large number of nodes, the adversary has no choice but to forge some MACs in order to fabricate a report. Such a report having forged MACs will be discarded by a forwarding node or rejected at a BS. Such a collaborative generation and en-route verification mechanism have been served as a foundation for providing resilience against FDIAs in other countermeasures [3–8]. In the interleaved hop-by-hop authentication scheme proposed by Zhu et al. [5], for example, a report must be endorsed by all the nodes in a cluster and verified by every forwarding node in an interleaved fashion. Polynomials instead of MACs can be used to endorse reports since it could improve resilience to forgeries of identities [22].

Li et al. [9] first found that such a mechanism has given rise to a vulnerability that a few compromised nodes can insert some false MACs into a report for a real event in order to suppress reporting of the event (i.e., FEIAs). Thus, they proposed PVFS that allows a report to be verified multiple times by forwarding nodes. Once the number of successful verifications has reached a predefined threshold value, the report is considered to be legitimate. The report is then forwarded to the BS without en-route verification, so that some resilience to FEIAs can be provided. If the number of failed verifications exceeds another threshold value, the report is dropped immediately in order to provide some resilience to FDIAs. MEF proposed by Kim and Cho [10] can be also used to defend FEIAs. In MEF, a forwarding node instantly drops a report if the verification fails, as in SEF. However, multiple copies of a report are generated for each event and are delivered to BSs through different paths. The keys used to endorse them differ from each other. Thus, the event can be reported to the users even if some of them were dropped by forwarding nodes. Another countermeasure proposed by Krauß et al. [11] enables a report generating node (e.g., a CH) to detect a node that has sent a false MAC, by making the node prove the correctness of the MAC. If the proof fails or the node does not perform the proof, the node is excluded in the report generation process. In [12], Yu et al. adopt polynomials instead of MACs for verification, which could increase resilience against forging identities.

All of these countermeasures are vulnerable to MCAs since they assume that a report generating node will compile a report with the MACs sent by other detecting nodes, without any manipulation. A compromised one, however, can compile a report for a real event with forged MACs although it has collected legitimate MACs from other detecting nodes. The real event will not be reported to the users since the report will be considered to be fabricated one representing nonexisting event.

## 6. Conclusions and Future Work

In this paper, the author proposed a method for detecting MCAs in cluster-based WSNs. In the proposed method, every report is collaboratively generated and verified by all nodes in a cluster based on very loose synchronization. Unless ⌈*N*/2⌉ or more nodes in a cluster are compromised, reports generated in that cluster could be delivered to the BSs. The performance of the proposed method was analyzed with analytical and experimental results. With only 10% overhead, the method could provide WSNs with MCA resilience. Therefore, it would be well worth considering for WSN security.

Although the method is designed for cluster-based WSNs, it could be employed for flat WSNs with a modification. Several issues not covered in the paper, including secure CH selections, CH-level collaboration, and slot assignment with the consideration of clock synchronization, will be studied. For the verification purpose, the author will try to formally prove the proposed method, for example, through model checking. In order to enhance the performance of the proposed method, the author will also try to apply other synchronization solutions or channel access methods to the method. By extending the method, en-route detection of report manipulation will be further investigated. All of these will be implemented on real nodes, in order to provide guidelines on the selection of design parameters, such as *N* and *T*_*S*_.

## Figures and Tables

**Table 1:** The sizes of the messages used to generate a report.

Size	The proposed method		The existing solutions	
	Not using key indices	Using key indices	Not using key indices	Using key indices
PSR	*S*_NID_ + *S*_*C*_ + 2 · *S*_MAC_	*S*_NID_ + *S*_*C*_ + *S*_KID_ + 2 · *S*_MAC_	*S*_NID_ + *S*_*C*_ + *S*_MAC_	*S*_NID_ + *S*_*C*_ + *S*_MAC_
EM	*S*_NID_ + 2 · *S*_MAC_	*S*_NID_ + *S*_KID_ + 2 · *S*_MAC_	*S*_NID_ + 2 · *S*_MAC_	*S*_NID_ + *S*_KID_ + 2 · *S*_MAC_
SR	*S*_CID_ + *S*_NID_ + *S*_*C*_ + *N* · *S*_MAC_	*S*_CID_ + *S*_NID_ + *S*_*C*_ + *N*(*S*_KID_ + *S*_MAC_)	*S*_CID_ + *S*_NID_ + *S*_*C*_ + *N* · *S*_MAC_	*S*_CID_ + *S*_NID_ + *S*_*C*_ + *N*(*S*_KID_ + *S*_MAC_)

**Table 2:** The number of MAC computations in the proposed method and the existing solutions.

MAC computations	The proposed method		The existing solutions	
	Random	Deterministic	Random	Deterministic
EAP	3 · *N* − 1	*N* + 1	2 · *N* − 1	*N *
REP	*N* ^2^ − 1	*N* ^2^ − 1	2 · *N* − 1	2 · *N* − 1
ERP	0	0	0	0
